# Microphysiological Systems of Lymphatics and Immune Organs

**DOI:** 10.1002/adhm.202503201

**Published:** 2025-09-29

**Authors:** Ishita Jain, Ankur Singh, Andrés J. García

**Affiliations:** ^1^ Woodruff School of Mechanical Engineering Georgia Institute of Technology Atlanta GA USA; ^2^ Petit Institute for Bioengineering and Biosciences Georgia Institute of Technology Atlanta GA USA; ^3^ Coulter Department of Biomedical Engineering Georgia Institute of Technology Atlanta GA USA

**Keywords:** hydrogels, lymph node, lymphadema, lymphatics, organ‐on‐chip

## Abstract

Microphysiological systems (MPS) that incorporate engineered blood vasculatures have enabled new opportunities to study human physiology and disease, offering platforms for drug development, tissue modeling, and regenerative medicine. However, most human tissues also contain an equally complex yet underrepresented secondary vascular network, the lymphatic system. Lymphatics play indispensable roles in interstitial fluid drainage, immune cell trafficking, and antigen presentation, and are central to the pathophysiology of diseases such as lymphedema, chronic inflammation, and cancer metastasis. Despite their critical biological functions, lymphatic vessels and associated immune structures, such as lymph nodes, remain absent from current in vitro models. Integrating lymphatics into biomaterials‐based MPS is essential for capturing the dynamic interplay between fluid transport, immune surveillance, and tissue homeostasis. This review surveys recent progress in engineering lymphatic microenvironments and immune organoids within biomaterials‐based MPS, emphasizing innovative strategies to recreate the biochemical and biophysical complexity of native lymphatic tissues. Advances are highlighted in tunable extracellular matrix platforms, humanized cell sourcing, and precision fabrication techniques, including perfusable, modular, and scalable models. The integration of lymphatic components with multi‐organ systems, combined with the application of computational modeling and machine learning, offers unprecedented opportunities to build personalized and physiologically relevant immune models. Incorporating lymphatics into next‐generation MPS promises to illuminate fundamental disease mechanisms and accelerate the development of more predictive therapeutic testing platforms with improved clinical translation.

## Graphical abstract

This review surveys recent progress in engineering lymphatic microenvironments and immune organoids within microphysiological systems, emphasizing innovative strategies to recreate the biochemical and biophysical complexity of native lymphatic tissues. Advances are highlighted in tunable extracellular matrix platforms, humanized cell sourcing, and precision fabrication techniques, including perfusable, modular, and scalable models.

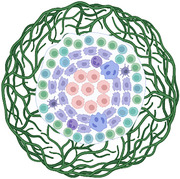

## 1 Introduction

Microphysiological systems (MPS) have emerged as powerful platforms for modeling human physiology and disease in controlled, modular environments. These engineered systems, often incorporating blood microvasculature, have advanced our understanding of tissue function, drug responses, and regenerative processes. However, a critical component of human vascular biology remains largely missing from current MPS designs: the lymphatic system. The lymphatic vasculature forms a secondary, complementary circulatory network that is essential for immune surveillance, fluid homeostasis, and the resolution of inflammation.^[^ ^1^, ^2^ ^]^ Lymphatic vessels collect interstitial fluid, termed lymph, from tissues and transport it through a hierarchical network of lymph nodes (400–800 in humans), where immune cells process antigens and initiate adaptive immune responses. The processed lymph is ultimately returned to the venous circulation,^[^ ^3^ ^]^ completing a one‐way transport loop that is indispensable to tissue health and systemic immunity.

Despite the central role of lymphatics in coordinating immune responses and maintaining physiological balance, their integration into in vitro models has lagged behind that of the blood vasculature. This gap is due, in part, to limited tools and biomimetic platforms that accurately capture lymphatic structure, transport dynamics, and immunological function. While many of these functions are well studied in vivo, recapitulating them in vitro remains a major challenge.

There is a pressing need to incorporate lymphatic networks and lymphoid structures into biomaterials‐based MPS to enable more physiologically relevant models of human disease, immunotherapy, and personalized medicine. This review highlights recent innovations in engineering lymphatic systems within MPS, with a focus on integrating lymphatic vessels and immune organoids. We discuss how these emerging platforms can unlock new insights into lymphatic biology and support the development of next‐generation tools for human health research.

## 2 Physiology of Lymph Nodes and Connecting Lymphatics

Lymph nodes are secondary lymphoid organs interconnected by afferent and efferent lymphatic vessels.^[^ ^2^ ^]^ Lymphatic capillaries, also known as initial lymphatics, are highly specialized, blind‐ended vessels composed of a single layer of loosely connected lymphatic endothelial cells (LECs).^[^ ^4^ ^]^ Unlike blood capillaries, these vessels are characterized by an absence of a continuous basement membrane and the lack of perivascular support cells, such as pericytes and smooth muscle cells, which contributes to their elevated permeability. A defining structural feature of these capillaries or initial lymphatics is the presence of discontinuous, button‐like intercellular junctions between LECs, which facilitate the uptake of interstitial fluid, macromolecules, immune cells, and even pathogens.^[^ ^5^ ^]^ These button junctions form specialized “primary valves” that open in response to increased interstitial fluid pressure in the surrounding tissue. Anchoring filaments tether the LECs to the extracellular matrix, enabling the vessels to sense mechanical cues and modulate valve opening accordingly.^[^ ^6^ ^]^ The inherent balance of fluid pressure and tissue elasticity is necessary in guiding lymphatic drainage.^[^ ^7^, ^8^ ^]^ The bioadhesivity and mechanical properties of the extracellular matrix (ECM), including stiffness, elasticity, and density, play a crucial role in regulating interstitial fluid dynamics and lymphatic vessel function.^[^ ^7^ ^]^ These properties influence how mechanical forces are transmitted through tissues, affecting lymph formation, vessel permeability, and lymphatic endothelial cell behavior. For example, while collagen fibers provide much of the structural integrity and tensile strength of tissues, glycosaminoglycans (GAGs) contribute to osmotic pressure regulation. Their high negative charge density causes them to repel one another and attract counterions, which increases local osmotic pressure and promotes water retention in the interstitial space, thereby influencing tissue hydration and lymph formation.^[^ ^7^ ^]^ The initial lymphatic capillaries drain the lymph into collecting lymphatic vessels which contains flap or secondary valves, ensuring unidirectional lymph flow by directing fluid from collecting vessels to primary and secondary lymphoid organs.^[^ ^9^ ^]^ The continuous filtration of plasma from the arterial end of blood capillaries into the interstitial space makes the efficient drainage function of lymphatic capillaries and vessels essential for maintaining tissue fluid balance and immune surveillance.

**Figure 1:**
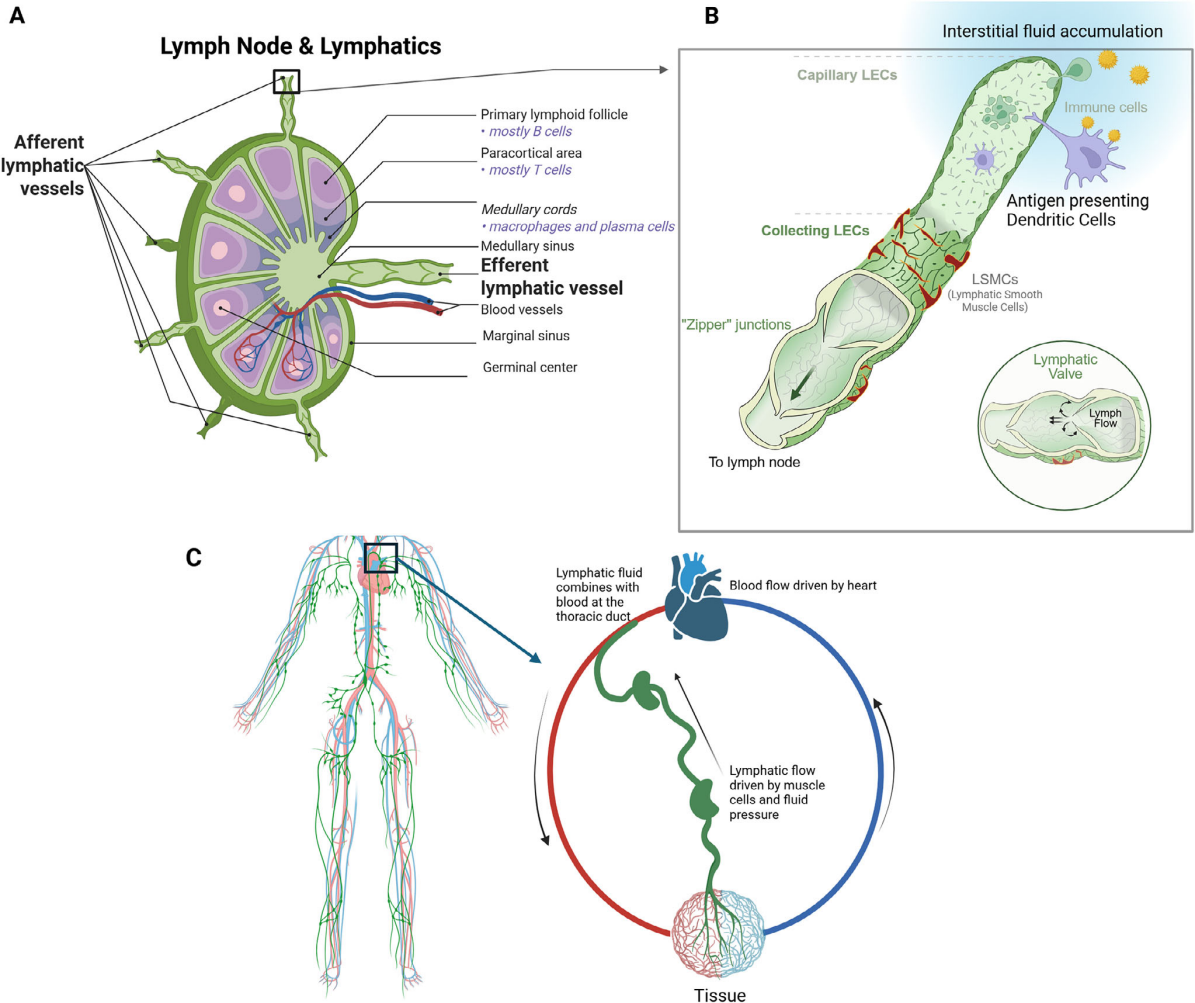
Schematic of Lymph Node Anatomy, Lymphatic Flow, and Circulation. A) The lymph node is a highly organized structure with distinct lobules of B cells and T cells. The lymph node receives lymph fluid from multiple afferent lymphatic vessels, which typically exit through a single major efferent lymphatic vessel. B) This panel illustrates the cellular structure of the lymphatics, beginning with the initial lymphatics in the tissue. Interstitial tissue fluid pressure guides the flow of fluid and antigen‐presenting immune cells into the lymphatics via loosely connected LECs. The cells and fluid flow unidirectionally toward the lymph node, aided by contractile smooth muscle cells and lymphatic valves. Lymphatic valves are zipper junctions inside the lymphatics that restrict the flow of lymph fluid away from the lymph node. C) Schematic of whole‐body lymphatic circulation and it's contrast to blood circulation. Blood circulation is bi‐directional, mediated by arteries (red) and veins (blue), with the heart being the master pump. Lymphatic circulation is a unidirectional flow of lymph going from peripheral tissue to lymph nodes to finally the thoracic duct for integration with the blood again. This flow is driven by interstitial fluid pressure and smooth muscle contractions and maintained unidirectional by lymphatic valves in the lymphatic vessels. Made with Biorender.

Afferent lymphatic vessels (**Figure** **1**) transport lymph carrying antigens and immune cells into the subcapsular sinus of lymph nodes, where initial immune surveillance occurs. From there, a portion of the lymph percolates through the inner cortex and medulla, facilitating interactions with resident immune cells, while the remainder flows along sinusoidal pathways. Approximately 90% of lymph entering a lymph node traverses the sinusoidal pathways, specifically through the subcapsular, cortical, and medullary sinuses, thereby facilitating rapid fluid transit. Conversely, only ≈5–10% of the lymph percolates through the parenchyma, comprising the inner cortex and medulla, where it encounters dense populations of B and T lymphocytes engaged in immune surveillance and antigen presentation. This selective flow mechanism balances the necessity for expedited fluid clearance with the requirement for effective immune processing. The precise proportion may vary depending on tissue type, inflammatory status, and the architectural characteristics of the lymph node.

Filtered lymph then exits via efferent lymphatic vessels, continuing to downstream lymph nodes or returning to the venous circulation. Multiple afferent vessels enter the lymph node, while filtered lymph then leaves via one or two efferent vessels, connecting to an efferent duct which flows the lymph back into the blood via lymph‐venous connections.^[^ ^10^ ^]^ The afferent and efferent lymphatic vessels perform distinct yet coordinated functions in immune homeostasis and response, with their cellular and molecular composition undergoing dramatic shifts under pathological conditions. Under physiological conditions, afferent lymph transports a low volume of migratory immune cells, predominantly antigen‐presenting dendritic cells (DCs), CD4⁺ T cells, and a limited number of B cells, into the lymph node. These CD4⁺ T cells display an activated phenotype, expressing markers including CD25 and MHC class II, and they produce cytokines such as IL‐2 and IFN‐γ.^[^ ^11^ ^]^ In efferent lymph, the majority of the output consists of naïve or resting CD4⁺ T cells and B cells that entered the lymph node through high endothelial venules (HEVs), indicating a key pathway for recirculation.^[^ ^12^ ^]^ Cytokine levels in efferent lymph are generally low, except for GM‐CSF.^[^ ^13^ ^]^

Upon encountering a pathological challenge, such as infection or antigen exposure, afferent lymph fluid undergoes distinct immunological remodeling. In early inflammation, neutrophils and DCs rapidly appear in afferent lymph, along with sharp increases in CCL21, IL‐1β, TNF‐α, and IL‐6 levels.^[^ ^14^ ^]^ This neutrophilic influx is transient, giving way to a surge in lymphoblasts, particularly activated CD4⁺ T cells that become the dominant producers of cytokines such as IL‐2, IFN‐γ, and GM‐CSF.^[^ ^15^ ^]^ These regulatory cytokines coordinate local immune responses by modulating the tissue microenvironment and augmenting the activity of other immune cells. Furthermore, these cytokines have been shown to directly influence LEC barrier function, with differential expansion of LEC cell‐cell junctions observed in response to various cytokines. For example, a 2.5‐fold increase in the length of LEC cell‐cell junctions was noted following exposure to IFN‐γ, potentially facilitating the recruitment of a larger number of immune cells to the lymphatic system.^[^ ^16^ ^]^ The IL‐17RC/CMTM4/NF‐κB axis in LECs further plays a role in clearing neutrophils from inflamed tissue and directing them to lymphatics.^[^ ^17^ ^]^ Interestingly, while the composition of afferent lymphocytes changes markedly, the flow rate and total cell output remain relatively constant during primary immune responses, with a few exceptions. Meanwhile, efferent lymph transitions through three well‐defined phases: an initial shutdown of lymphocyte exit, a recruitment phase characterized by increased lymphocyte trafficking and emergence of non‐recirculating effector blasts, and a resolution phase, where cell trafficking normalizes. The effector T and B lymphoblasts in efferent lymph do not recirculate but migrate to inflamed tissues, representing the outcome of antigen‐specific activation.^[^ ^10^ ^]^ Together, these phenomena emphasize the role of afferent lymph as a sensor and signaler of tissue pathology, and of efferent lymph as a distributor of immune effectors, highlighting a highly regulated, compartmentalized immune response that varies between steady‐state surveillance and active immune defense.^[^ ^18^ ^]^

The lymphoid lobule is the fundamental functional unit of the lymph node, structured around a delicate reticular meshwork of fibroblastic reticular cells (FRCs) and their fibers.^[^ ^19^ ^]^ This meshwork creates narrow channels filled with lymphocytes, macrophages, and antigen‐presenting cells (APCs), providing a 3D scaffold for the migration and interaction of immune cells. Lymphocytes, the parenchymal cells of the lobule, continually recirculate through HEVs into the lobules and exit via paracortical sinuses into efferent lymph. HEVs are specialized cuboidal endothelial vessels that control lymphocyte entry and respond to inflammatory signals carried by lymph through a conduit system linked to the reticular meshwork. This interconnected network enables lymph nodes to act as “information marketplaces,” where APCs present antigenic information and lymphocytes search for specific pathogens. The lymph node is functionally and spatially organized, with B and T cells residing in distinct lobular areas, undergoing clonal expansion upon encountering antigens.^[^ ^20^ ^]^ Every lobule gets lymph from its own unique afferent vessels, exposing it to a range of antigenic stimuli. This leads to different immune responses across the node. The lobule's complex structure and cell traffic allow for effective systemic immune surveillance and response.^[^ ^2^ ^]^ One of the main characteristics of lymph nodes is the germinal centers (GC), which are local B cell clusters undergoing proliferation and somatic hypermutation to build antigen‐specific antibody‐producing B cells. GCs are often a result of antigen exposure and part of the adaptive immune response that also leads to differentiation of GC B cells into memory B cells for later recall.

After entering the lymph node via the subcapsular sinus, lymph flows through a highly specialized microenvironment that is not only defined by its cellular composition but also by its distinct biophysical and biochemical properties, which dynamically respond to immune challenges. For instance, the lymph node exhibits strain‐stiffening behavior, with its Young's modulus increasing from ≈50 kPa at 30% strain to 300 kPa at 100% strain. This strain hardening reflects the tissue's ability to mechanically adapt to increasing physiological stresses, such as cellular influx during inflammation.^[^ ^21^ ^]^ In addition to its mechanical properties, the lymph node is rich in ECM components, particularly within the reticular network formed by fibroblastic reticular cells (FRCs). Key ECM proteins in this network include collagens I, III, and IV, tenascin, laminin, and fibronectin, all of which contribute to the structural organization and functional regulation of immune cells.^[^ ^22^ ^]^ Notably, ECM remodeling plays a critical role in multiple key immune regulation pathways such as inflammation, immune cell trafficking, and lymphatics remodeling.^[^ ^23^, ^24^ ^]^ For example, during inflammation, remodeling of laminin in the resting versus the inflamed lymph node has been linked to T cell differentiation.^[^ ^24^ ^]^ High expression of laminin α5 is associated with proinflammatory responses, while laminin α4 is more prominent in tolerogenic lymph node environments.^[^ ^25^, ^26^ ^]^ Furthermore, upon inflammation, there is increased proliferation of FRCs and other stromal cells in the lymph node, leading to substantial remodeling of the microenvironment and changes in ECM composition that contribute to LN expansion.^[^ ^24^ ^]^

## 3 Disease Relevance and the Need for Lymphatics‐Integrated In Vitro Systems

**Figure 2:**
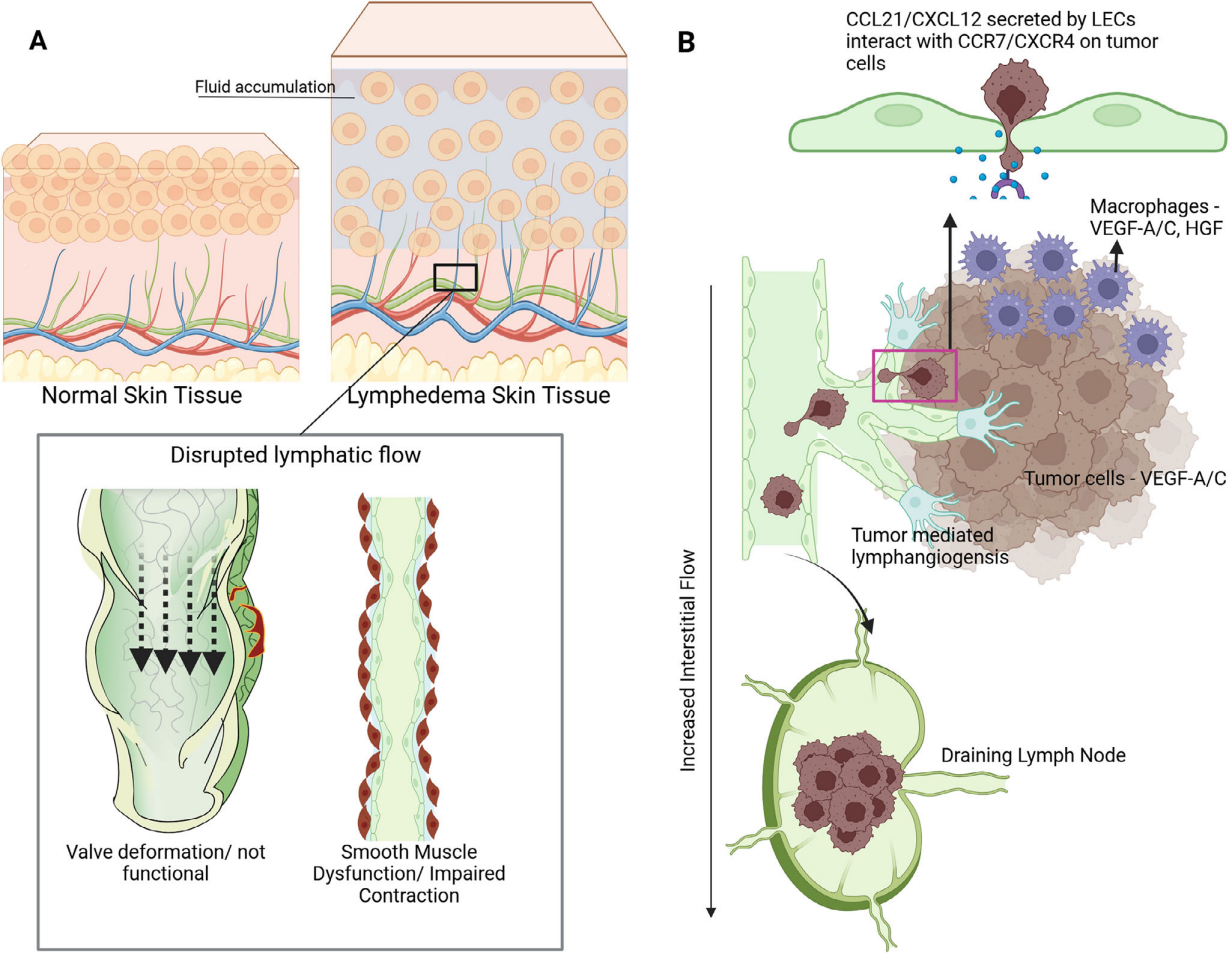
Role of Lymphatics in Disease Relating to Immune Systems. A) Fluid accumulation is demonstrated in the skin tissue of a healthy patient and a patient with lymphedema. Due to a disruption in lymphatic flow, fluid begins to accumulate in peripheral tissues, causing swelling in the hands and legs. This disruption could be due to valve malformations or loss of muscle contractility in the lymphatics. B) Cancer metastasis has been linked to increased proliferation of LECs, leading to a higher density of lymphatic vessels at tumor sites due to VEGF‐A/C secreted by surrounding macrophages and/or tumor cells. This enables the intravasation of tumor cells into the lymphatics via CCR7/CXCR4 receptors and leads to the migration of cancer cells to draining lymph nodes, leading to further metastasis to other tissues.^[^ ^40^ ^]^ Made with Biorender.

Multiple pathophysiological conditions result from disruptions in primary or secondary lymphatic function.^[^ ^27^, ^28^ ^]^ Lymphedema is the accumulation of interstitial fluid in tissues caused by impaired lymphatic vessel function (**Figure** **2**A), which disrupts the normal drainage of lymph.^[^ ^29^ ^]^ Primary lymphedema arises from congenital or developmental abnormalities in the lymphatic system, which impair the proper drainage of lymph. Secondary lymphedema is caused by damage to lymphatic vessels from trauma, surgery, cancer treatment, or infections. Damage to the lymphatic system can lead to issues with lymph transport due to faulty valves, abnormal lymphatic vessel development, or reduced muscle movement. Clinically, lymphedema is characterized by persistent swelling, localized pain, skin changes such as thickening and fibrosis, and an increased risk of infections due to weakened immune function. The swelling and disfigurement caused by lymphedema can have a major impact on the quality of life of patients, resulting in psychological distress and social stigma.^[^ ^30^ ^]^ One of the most common and well‐studied types of secondary lymphedema is breast cancer‐related lymphedema, which happens after lymph nodes are removed during cancer treatment.^[^ ^31^ ^]^ Recapitulating lymphedema in vitro with precise temporal and spatial modelling of lymphatic dysfunction is crucial for a deeper understanding of the molecular pathology of lymphedema. Furthermore, the effects of microenvironmental factors such as ECM remodeling, fibrosis, and mechanical properties in regulating lymphedema remain understudied.

While lymphatic dysfunction contributes to fluid accumulation and immune dysregulation in lymphedema, abnormal lymphatic remodeling also plays a role in cancer progression. In particular, tumor‐associated lymphangiogenesis, driven by vascular endothelial growth factors such as VEGF‐A and VEGF‐C, has been strongly linked to metastasis (Figure 2B) in cancers including melanoma, breast, and lung malignancies.^[^ ^32^ ^]^ Increased lymphatic vessel density and enlargement in tumors are linked to metastasis to draining lymph nodes, distant spread, and poor prognosis. Tumors primarily promote lymphangiogenesis through the proliferation and sprouting of existing lymphatic vessels, rather than recruiting circulating endothelial progenitor cells; however, some bone marrow‐derived macrophages may also contribute to this process. In murine models, VEGF‐A and VEGF‐C promote lymphangiogenesis not only at the tumor site but also in tumor‐draining lymph nodes before metastatic cells arrive, creating a favorable environment for cancer spread.^[^ ^33^ ^]^ This pre‐metastatic lymph node lymphangiogenesis has been observed in melanoma and breast cancer models, as well as in human patients, where increased lymphatic growth in metastatic lymph nodes correlates with further metastasis. Overall, tumor‐induced lymphangiogenesis in both primary tumors and draining lymph nodes facilitates tumor dissemination through the lymphatic system.

Lymph node transplantation and engineered lymphatic grafts for the treatment of lymphedema have shown promise but remain limited by a lack of mechanistic understanding and standardization.^[^ ^34^, ^35^, ^36^ ^]^ Most commonly, a healthy lymph node from a different site is used for a lymph node transplant in the same patient to treat lymphedema; however, this yields limited results in improving lymphatic drainage and can lead to complications such as donor‐site morbidity.^[^ ^37^ ^]^ Furthermore, a deeper understanding of regained immune function and lymphatic drainage is lacking. There is a need to have a comprehensive understanding of these disease mechanisms, recapitulate them in vitro and in vivo, and optimize these engineered grafts accordingly. in vitro models mimicking native tissue biology, architecture, and biological properties^[^ ^38^, ^39^ ^]^ offer controlled platforms to investigate these diseases, develop therapies, and test immune interventions with human‐relevant physiology.

## 4 in vitro Models of Lymphatics

In vitro models have been instrumental in advancing our understanding of the lymphatic system, particularly lymphangiogenesis, the formation of new lymphatic vessels from pre‐existing ones, analogous to angiogenesis. Most studies of lymphangiogenesis rely on 2D cultures or modified 3D angiogenesis assays, commonly using ECM such as Matrigel, collagen, or fibrin. Whereas Matrigel is widely adopted due to its ease of use and bioactivity, its utility is limited in applications requiring precise control over biophysical and biochemical properties that can drastically influence LEC phenotype and function.^[^ ^41^, ^42^ ^]^ Additionally, significant batch‐to‐batch variability in Matrigel composition introduces experimental inconsistencies, which complicate data interpretation and reproducibility. These limitations underscore the need for well‐defined, tunable biomaterials that provide greater control and standardization for in vitro lymphatic research.

Advances in tissue‐engineered models using well‐defined biomaterials have significantly enhanced our ability to replicate key aspects of human tissue physiology and pathology in vitro. Engineered systems can recapitulate lymphatic capillary networks in vitro, providing new insights into lymphangiogenesis and lymphatic remodeling. Researchers generally utilize LEC cellular protein expression such as podoplanin (PDPN), the master transcription factor PROX1, and lymphatic vessel endothelial hyaluronan receptor‐1 (LYVE‐1), to systematically evaluate lymphatic phenotype and morphology. Among these, hydrogels have emerged as particularly versatile platforms, offering biocompatibility and tunable mechanical and biochemical properties suitable for 3D cell culture. Natural hydrogels such as collagen and fibrin are especially well‐suited for vascular applications, supporting endothelial tube formation and enabling the establishment of biochemical gradients. Notably, Marino et al. developed a skin graft model incorporating human dermal microvascular endothelial cells, including both blood (BECs) and lymphatic (LECs) endothelial populations, within a collagen–fibrin hydrogel scaffold. When implanted into immunocompromised rats, these grafts facilitated the formation of functional lymphatic and blood capillaries that successfully integrated with the host vasculature, thereby enhancing interstitial fluid drainage. This outcome underscores the potential of biomaterial‐based platforms to support lympho‐vascular regeneration in vivo.^[^ ^43^ ^]^

Gibot et al.^[^ ^44^ ^]^ developed a cell‐based, biomaterial‐free platform in which human LECs co‐cultured with fibroblasts self‐assembled into stable 3D lymphatic networks. This process was driven by fibroblast‐secreted VEGF‐C and hepatocyte growth factor (HGF), resulting in structures that displayed key molecular and morphological features of native lymphatic vessels. Optimizing multiple parameters—including collagen/fibrin composition, growth factor concentrations, and LEC‐to‐fibroblast ratios—was essential to achieving robust network formation. In parallel, the role of mechanical cues in guiding lymphatic morphogenesis has also come into focus. Landau et al. applied cyclic mechanical stretch (20% strain for 21 days) to fibrin‐based constructs, which promoted the alignment of lymphatic vessels within engineered muscle tissue.^[^ ^45^ ^]^ Complementary studies have revealed that matrix composition and biophysical context distinctly regulate capillary formation by blood and lymphatic endothelial cells: while blood endothelial cells (BECs) favored collagen‐rich environments and formed thick, branched vessels, LECs formed finer, elongated structures within fibrin‐only matrices under interstitial flow (≈4.5 µm s^−1^).^[^ ^46^ ^]^ Furthermore, Knezevic et al. demonstrated that co‐culturing with adipose‐derived stem cells and graded concentrations of VEGF‐C induced dose‐dependent lymphatic network formation in fibrin hydrogels.^[^ ^47^ ^]^ Collectively, these studies highlight that fibrin (2.5–15 mg mL^−1^) and collagen (0.5–3 mg mL^−1^) matrices, when combined with VEGF‐C (25‐100 ng mL^−1^), support lymphatic capillary formation both in vitro and in vivo. Importantly, the integration of biomechanical forces—such as cyclic strain and controlled interstitial flow—emerges as a critical regulator of lymphatic morphogenesis, underscoring the multifactorial nature of engineered lymphatic model development (**Table** **1**).

Use of natural ECMs such as collagen I and fibrin for engineered lymphatics has demonstrated the potential of hydrogels, but also provides limited control on LEC phenotype and functionality, where well‐defined functional long‐term vessel networks have not been achieved. Over the past decade, the biomaterials field has advanced significantly in developing tunable synthetic hydrogels for a wide range of applications in studying human physiology and diseases. Synthetic hydrogels offer precise control over various biomaterial properties, such as stiffness, ligand presentation, stress‐relaxation, and can be made dynamic.^[^ ^48^ ^]^ Early demonstrations of lymphatic grafts utilizing synthetic hydrogels involved LECs seeded into polyglycolic acid (PGA) scaffolds and implanted in mice.^[^ ^49^ ^]^ Alternatively, using a polyethylene glycol (PEG)‐ based hydrogel presenting VEGF‐C, researchers found that optimal lymphatic sprouting from rat collecting vessels occurred in a matrix with a stiffness of 680 Pa, full protease degradability, and a 2.0 mm RGD (Arg‐Gly‐Asp) peptide concentration. This hydrogel‐supported functional vessel grafting offers a promising tool for in vivo lymphangiogenesis and mechanistic studies.^[^ ^39^ ^]^ Alderfer et al. optimized a multi‐parameter hyaluronic acid (HA) hydrogel for in vitro and in vivo lymphatic vessel formation.^[^ ^48^ ^]^ The study reports that 25% crosslinked norbornene‐modified (HA) hydrogel, with 5 mm RGD and 50 ng mL^−1^ VEGF‐C, resulted in a well‐defined lymphatic capillary network in vitro. Fan et al. developed a UV‐tunable hyaluronic acid hydrogel to spatially control viscoelasticity without complex chemical processing, mimicking native ECM dynamics. This system enhanced the formation of lymphatic cord‐like structures by LECs through the upregulation of key lymphatic markers and VEGF‐C signaling pathways.^[^ ^50^ ^]^ Overall, the field is advancing toward the development of multiple synthetic hydrogel systems for creating lymphatic capillary networks. There is great potential in advancing the field toward the integration of these networks with other tissues and in advancing these models to study diseases like lymphedema.

Hydrogel‐based platforms have significantly advanced the development of in vitro lymphatic models by offering tunable, cytocompatible environments that support lymphangiogenesis. Nonetheless, replicating the full physiological complexity of native lymphatic tissues remains a major challenge. Existing systems have limited capabilities when it comes to structural organization LECs (initial vs collecting), long‐term stability, functional lumen formation, and intraluminal valve development. Moreover, although both natural and synthetic hydrogels can be engineered to present specific mechanical and biochemical signals, we speculate that their design is often optimized for angiogenesis and may not recapitulate the distinct biological and mechanical requirements of the lymphatic vasculature. The ongoing refinement of hydrogel design to facilitate long‐term fluid drainage via valves and inter‐connection of multiple tissues will facilitate advancements in both fundamental understanding and translational applications in lymphatic biology and regenerative medicine.

**Figure 3:**
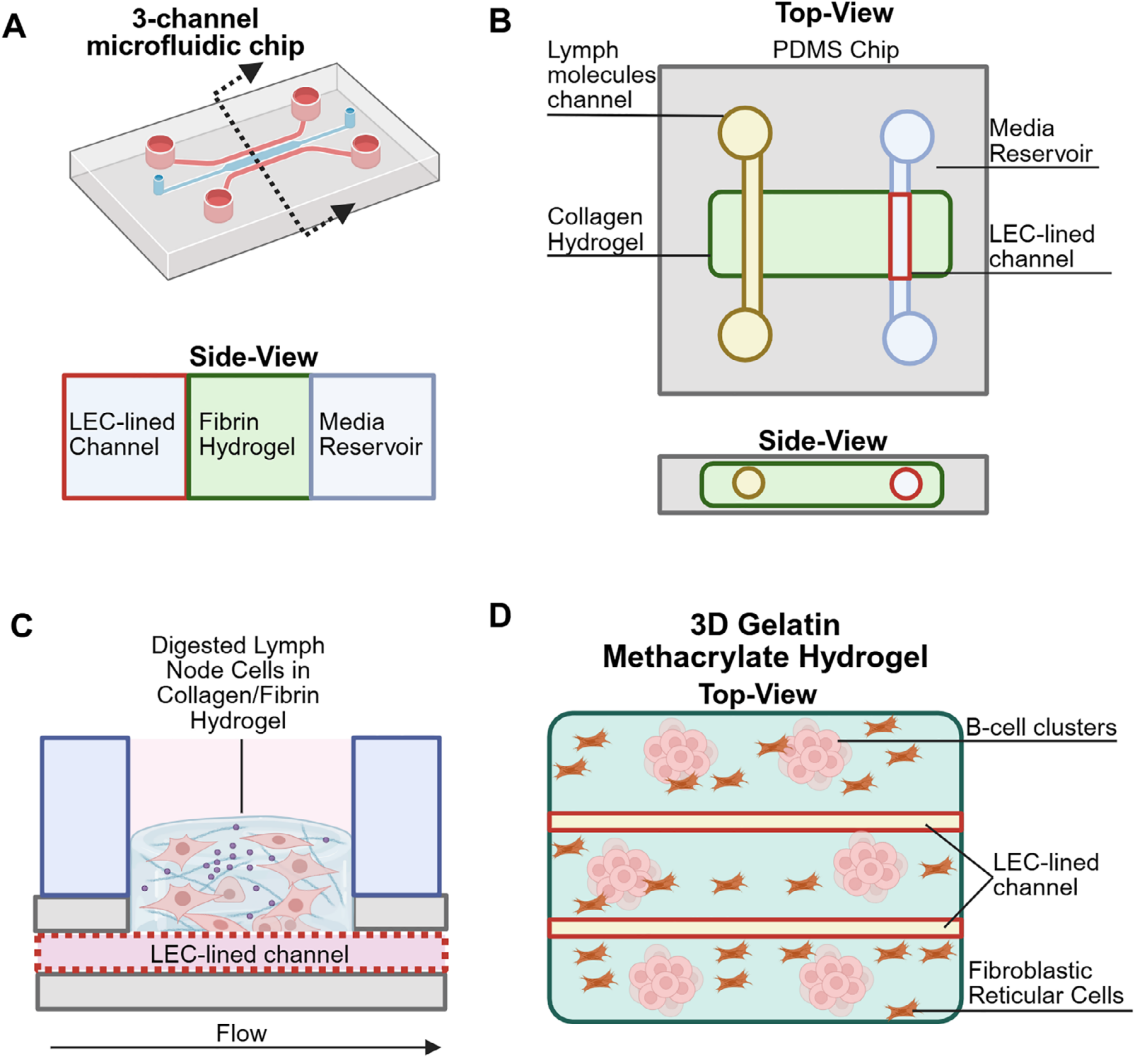
Representative schematics of specific MPS with lymphatics and immune cells. A) Adapted from Serrano et al.^[^ ^57^ ^]^ a 3D PDMS microfluidic chip with 3 channels. LECs were lined in one, followed by a fibrin hydrogel filled channel in the middle and a media channel at the end. This system enabled evaluating lymphangiogenesis of LECs into the fibrin hydrogel and also model drainage. B) Adapted from Lee et al.^[^ ^56^ ^]^ a 3D PDMS chip with a collagen hydrogel in the center. Through the hydrogel, 2 hollow channels were fabricated, one lined with LECs and the other for flowing lymph molecules such as fatty acids or albumin. LEC capillary formation through collagen hydrogel was observed, and drainage of the lymph molecules through the hydrogel into the LEC channel lumen was quantified. C) Adapted from Morrison et al.^[^ ^87^ ^]^ a organ‐on‐chip model of human lymph node with perfused lymphatics is developed. This system consisted of an LEC lined channel with human digested lymph node cells in a collagen/fibrin hydrogel on top. D) Adapted from Mazzaglia et al.^[^ ^86^ ^]^ a 3D gelatin‐methacrylate hydrogel was developed with 2 hollow channels lined with LECs. The hydrogel composed of lymph node derived fibroblasts and B‐cell clusters to study the interaction of immune cells and lymphatics. Schematics made with Biorender.

Microfluidic systems have emerged as powerful platforms for lymphatic tissue engineering, offering the ability to replicate physiologically relevant interstitial and luminal shear forces while supporting co‐culture with other tissue‐engineered components. These systems can be leveraged for drug development and mechanistic studies, with the added potential for higher‐throughput screening formats. For example, microfluidic devices incorporating interstitial flow (≈1–2 µm s^−1^) across lymphatic fibrin grafts have been shown to promote lymphangiogenesis and enhance barrier function.^[^ ^51^ ^]^ A lymphatic vessel‐on‐chip model, constructed using human LECs embedded within a 3D hydrogel microchannel, enabled real‐time assessment of lymphatic permeability under controlled luminal shear flow (3.5–4.5 dyn cm^−^^2^). This model revealed that fibronectin strengthens LEC junctions and reduces permeability through activation of integrin α5, a key regulator of lymphatic barrier integrity.^[^ ^52^ ^]^ Despite these advances, current engineered lymphatic vessels exhibit permeability levels (10 × 10^−6^ cm s^−1^) that remain an order of magnitude higher than physiological lymphatic capillaries (1‐6 × 10^−7^ cm s^−1^), underscoring the need for further refinement. Physiological shear stress in lymphatics ranges from 0.1‐10 dyn cm^−2^, exhibiting a wide range across spatial and temporal scales.^[^ ^53^, ^54^ ^]^ There is a need to build dynamic systems with oscillating shear flow to recapitulate physiological and pathological conditions. Disease‐relevant lymphatic models have also yielded new insights. Colon tumor–lymphatic co‐culture systems have been utilized to investigate cancer cell transmigration, lymphatic permeability, and immune modulation; however, detailed mechanistic studies of lymphatic involvement in metastasis are still lacking.^[^ ^55^ ^]^ Advanced platforms now integrate perfused lymphatic vessels with blood vasculature, enabling multi‐compartment models that simulate immune cell migration, antigen transport, and inflammation resolution (Figure 3B).^[^ ^56^ ^]^ For instance, Serrano et al.^[^ ^57^ ^]^ developed a microfluidic lymphatic‐on‐chip system that recapitulates in vivo lymphangiogenesis, lymph protein drainage, and immune cell recruitment during inflammation. This platform provides a versatile tool for probing lymphatic function and offers a foundation for therapeutic screening in lymphatic‐associated diseases (**Figure** **3**A). Overall, microfluidic systems have incorporated key features such as physiologically relevant flow, permeability assays, and co‐culture with stromal or immune cells. These models provide versatile tools for probing lymphatic function and therapeutic screening. Incorporating dynamic oscillatory flow and pressure gradients characteristic of native lymphatics^[^ ^53^, ^54^ ^]^ will further enhance physiological relevance. Such advances will enable deeper insights into lymphatic biology and accelerate the development of therapies for lymphatic‐associated diseases.

**Table 1:** Summary of key developments in engineering of in vitro lymphatics models.

Category	Materials / Systems Used	Cell Sources	What Was Modeled / Key Findings	Phenotypes Achieved
Natural Hydrogels	Fibrin Hydrogel + Interstitial Flow^[^ ^46^ ^]^	Human LECs	Fibrin and Interstitial flow supported vessel network formation	3D vessel network
	Collagen–Fibrin hydrogel^[^ ^43^ ^]^	Human dermal microvascular ECs (BECs + LECs)	Skin graft model → functional lymphatic & blood capillaries; host integration.	Host anastomosis, fluid drainage, capillary morphology in vivo
	Fibrin + cyclic stretch^[^ ^45^ ^]^	Human LECs	Cyclic strain aligned lymphatic vessels in engineered muscle.	Vessel alignment, morphogenesis under strain.
	Fibrin^[^ ^47^ ^]^	Human LECs + adipose‐derived stem cells	VEGF‐C induced dose‐dependent lymphatic networks.	3D vessel network
Biomaterial‐Free	LECs + Fibroblasts^[^ ^44^ ^]^	Human LECs + fibroblasts	Fibroblast‐secreted VEGF‐C/HGF drove self‐assembly into 3D lymphatic networks.	3D vessel network
Synthetic Hydrogels	PGA scaffolds bound VEGF‐C^[^ ^49^ ^]^	Human LECs	Implanted in mice; early synthetic lymphatic grafts.	Graft integration, vessel formation in vivo.
	PEG hydrogel + RGD^[^ ^39^ ^]^	Rat collecting vessel LECs	Optimal sprouting at 680 Pa, degradable, 2.0 mM RGD; functional grafting.	Lymphatic sprouting, vessel integration in vivo.
	HA hydrogel^[^ ^48^ ^]^	Human LECs	Crosslinked HA (RGD) supported robust lymphatic capillary networks.	Capillary density, network morphology.
	UV‐tunable HA hydrogel^[^ ^50^ ^]^	Human LECs	Dynamic viscoelasticity → enhanced cord‐like lymphatic structures.	3D vessel network with cord‐like structures
Microfluidic Systems	Fibrin grafts under interstitial flow^[^ ^51^ ^]^	Human LECs	Promoted lymphangiogenesis and barrier function.	3D vessel formation, barrier integrity.
	Lymphatic vessel‐on‐chip (Collagen 1 microchannel)^[^ ^52^ ^]^	Human LECs	Enabled permeability assays; fibronectin reduced permeability.	Permeability (10⁻⁶ cm/s range), junction integrity, and integrin α5 activation.
	Colon tumor–lymphatic co‐culture^[^ ^55^ ^]^	Mouse Tumor Cells + immortalized Human LECs	Modeled cancer cell transmigration, lymphatic permeability, and immune modulation.	Tumor transmigration, permeability, and immune interactions.
	Perfused lymphatic + blood vasculature^[^ ^57^ ^]^	LECs + BECs + immune cells	Simulated immune migration, antigen transport, and inflammation resolution.	Immune recruitment, antigen clearance, and dynamic lymphangiogenesis.

## 5 Engineering of in vitro Functional Immune Models

Over the past decade, major breakthroughs have been made in creating immune organoids that mimic the functions of lymphoid tissue, including T and B cell zones, antigen presentation, immune cell development, and dysregulation,^[^ ^58^, ^59^, ^60^, ^61^, ^62^, ^63^, ^64^, ^65^, ^66^, ^67^, ^68^, ^69^, ^70^, ^71^, ^72^ ^]^ although some of these represent simple aggregates of cells without biomaterials and a lymphoid niche.^[^ ^69^ ^]^ Studies have focused on replicating biological processes in different types of lymphoid tissues, such as bone marrow, thymus, tonsils, lymph nodes, and spleen.^[^ ^73^ ^]^ These engineered platforms of immune organs hold great potential to be integrated with engineered lymphatic vessel networks to study key processes, such as but not limited to vaccine trafficking, early immune cell development, and efficacy of immunotherapy.^[^ ^74^ ^]^ Lymphatic circulation and immune crosstalk across different lymphoid organs are extremely important in vaccine/immunotherapy development. Understanding the breakthroughs in recapitulating the different lymphoid organs is imperative to build functional integrated systems with lymphatics.

Recent advances in engineering primary lymphoid organs have enabled the development of bone marrow and thymic organoids that recapitulate essential processes of hematopoiesis and T cell development. Khan et al. developed a 3D bone marrow organoid from human iPSCs that closely mimics native bone marrow architecture, supporting vascular and hematopoietic lineage development. Incorporating VEGF‐C enhanced expression of bone marrow‐specific endothelial markers and key signaling pathways, reflecting native regulatory interactions. These organoids enable modeling of hematopoiesis and cancer‐stroma interactions.^[^ ^75^ ^]^ Emerging strategies have also led to the development of artificial human thymic organoids using hematopoietic stem progenitor cells (HSPCs) and DLL1‐transduced stromal cells, enabling positive selection of T cells via MHC‐presenting dendritic cells and HSPCs.^[^ ^76^ ^]^ These organoids show promise for generating TCR‐engineered T cells for immunotherapy. Efforts to incorporate functional T cells and address HLA polymorphisms are critical for improving model fidelity and clinical relevance. Stem cell‐derived T cells from iPSCs offer a scalable, patient‐specific alternative, bridging gaps in immunological modeling and regenerative medicine.^[^ ^77^, ^78^ ^]^ Although lymphatic capillaries have been visualized in the thymus in several studies,^[^ ^79^, ^80^ ^]^ their specific functional roles within this primary lymphoid organ remain poorly understood. Despite current progress in engineering of bone marrow and thymic organoids, they do lack integration with lymphatic structures, leaving a critical gap in fully modeling immune cell trafficking and cross‐talk within primary lymphoid environments.

More broadly, efforts to recapitulate secondary lymphoid organ function in vitro have led to promising yet technically challenging models. Moeller et al.^[^ ^59^ ^]^ reported a 3D biomaterials‐based B cell follicular organoid system to model germinal center (GC)‐like responses in vitro. Upon exposure to glycoengineered antigen–carrier conjugates, the organoids exhibited hallmark features of GC activation, including B cell receptor clustering, intracellular signaling, and somatic hypermutation. The immunogenicity of the conjugates modulated these responses and closely mirrored the magnitude of humoral responses observed in vivo. When combined with Next‐Generation Sequencing and Yeast Display analysis, this platform notably enabled the rapid identification of high‐affinity antibodies against conjugate components, significantly accelerating the timeline compared to conventional animal immunization workflows. These findings underscore the potential of synthetic lymphoid organoids not only as predictive tools for evaluating vaccine efficacy but also as platforms for the expedited discovery of antigen‐specific antibodies.

Wagar et al. developed an immune organoid system by reaggregating cryopreserved human tonsil cells, successfully recapitulating features of adaptive immunity, including B cell maturation and antibody class switching.^[^ ^71^ ^]^ However, this approach faced limitations in reproducibility and scalability, highlighting the need for more standardized and tunable platforms to study human immune and lymphatic interactions. Additionally, encapsulating tonsil epithelial cells in Matrigel™ and forming them into matrix structures has led to organoids that naturally respond to the immune system and are susceptible to SARS‐CoV‐2, making them ideal for testing antivirals. Some early versions of engineered lympho‐organoids, created from mouse stromal progenitor cells and ECM scaffolds, can help restore immune function in removed lymph nodes, although they have some limitations specific to the species.^[^ ^36^ ^]^

The tonsil aggregates,^[^ ^71^ ^]^ as well as those reported by others,^[^ ^81^ ^]^ represent the formation of memory B cell response, and largely lack the evidence of de novo antibody formation. Jeger‐Madiot et al. developed a lymphoid organ‐chip using a microfluidic system with human PBMCs encapsulated in a 3D collagen hydrogel to model vaccine responses.^[^ ^81^ ^]^ Perfusion with SARS‐CoV‐2 spike protein induced antigen‐specific CD4+ T cell and B cell clusters, as well as differentiation into plasma blasts. Furthermore, the lymphoid cells in the chip were competent at capturing and expressing mRNA vectored by lipid nanoparticles, enabling the assessment of responses to mRNA vaccines.

To overcome these limitations, synthetic hydrogel platforms engineered to mimic the lymphoid tissue microenvironment have enabled the formation of functional human germinal centers (GCs) from both tonsillar and peripheral blood mononuclear cell (PBMC)‐derived B cells. Zhong et al.^[^ ^67^ ^]^ engineered human immune organoids, focusing on the complex maturation processes of B and T cells, and mimicked germinal center (GC) responses using precisely engineered hydrogels with controlled stiffness and matrix composition.^[^ ^67^ ^]^ The parameters of the hydrogel were systematically optimized using spatial multi‐omics and atomic‐force microscopy‐based measurement of lymphoid tissue mechanical properties, to capture various biological processes of the B cell follicles. They demonstrated donor responses and adjuvant testing, along with the spatial organization of B cells, as seen in lymph nodes. Notably, immune organoids generated from PBMCs sustain GC B cells and plasma cells for longer durations than those derived from tonsils, and exhibit robust B cell programming, including the formation of distinct GC‐like compartments, somatic hypermutation, immunoglobulin class switching, and clonal expansion. The modulation of transcriptional and epigenetic pathways has been shown to further enhance plasma cell differentiation in these systems. This study presents the first evidence of inducing de novo GC and antibody responses, starting with memory‐depleted naïve B cells, and does not rely on the availability of antigen‐specific T cells. Moreover, spatially organized presentation of chemokines, such as polarized CXCL12 within a lymphoid organ‐on‐chip, can fine‐tune GC responses in healthy donor‐derived B cells.^[^ ^67^ ^]^ However, this response is markedly impaired in B cells derived from patients with lymphoma, underscoring disease‐specific defects in microenvironmental sensing and signaling.^[^ ^67^ ^]^ This demonstrated the potential of such engineered systems to recapitulate key immune pathways relying on spatial organization. Ongoing efforts aim to integrate such platforms with other tissue‐engineered systems to study immune dynamics in both homeostatic and disease contexts, including cancer, autoimmunity, and infection.

Whereas engineering of primary and secondary lymphoid organoids has yielded platforms capable of modeling hematopoiesis, T cell maturation, and germinal center responses (**Table** **2**), several limitations remain. Many systems rely on simplified cell aggregates or non‐tunable components that fail to fully recapitulate the stromal and vascular complexity of native lymphoid tissues. Reproducibility and scalability also remain challenges, as seen with tonsil‐derived aggregates, which limit standardization across studies. Importantly, most current models omit integration with functional lymphatic vessels, thereby excluding the critical roles of lymphatic circulation in antigen transport, immune cell trafficking, and inter‐organ communication. Together, these shortcomings highlight the need for next‐generation platforms that couple biomaterial engineering with lymphatic and vascular integration to achieve physiologically relevant immune modeling. Looking ahead, integrating lymphatic networks with engineered lymphoid organoids offers a powerful strategy to bridge these gaps and establish holistic systems for studying immunity in both health and disease.

**Table 2:** Summary of key developments in the engineering of functional in vitro immune models.

Material	Cell Sources	What was Modelled	Functions Achieved
60% Col I and IV, 40% Matrigel^[^ ^45^, ^75^ ^]^	Human iPSCs	Bone marrow organoid	Hematopoiesis, vascular/hematopoietic lineage development, cancer–stroma interactions
Cell Aggregates^[^ ^76^ ^]^	Human HSPCs, DLL‐1 induced Stromal cells	Thymus organoid	Positive selection of T cells, generation of TCR‐engineered T cells for immunotherapy
PEG‐based hydrogel^[^ ^59^ ^]^	Mouse B cells	Germinal center (GC)‐like organoid	BCR clustering, somatic hypermutation, antibody discovery, vaccine efficacy prediction
Transwell Cell Aggregates^[^ ^71^ ^]^	Cryopreserved human tonsil cells	Tonsil organoid	B cell maturation, antibody class switching, adaptive immune features
3D collagen hydrogel, microfluidic chip^[^ ^81^ ^]^	Human PBMCs	Lymphoid organ‐chip	Antigen‐specific CD4+ T/B clusters, plasmablast differentiation, mRNA vaccine response
PEG‐based hydrogel^[^ ^67^ ^]^	Human PBMCs	Germinal center (GC) immune organoid	Sustained GC B cells and plasma cells, somatic hypermutation, class switching, de novo antibody responses, donor‐specific/adjuvant testing
Lymphoid organ‐on‐chip with polarized chemokine gradients^[^ ^67^ ^]^	Human B cells (healthy and lymphoma patients)	GC microenvironment with spatial organization	Fine‐tuned GC responses revealed disease‐specific signaling defects

## 6 Multi‐Tissue Platforms of Immune Organoids and Lymphatic Networks

To explore the interplay between immune function and lymphatic biology, this section reviews recent advances in multi‐tissue platforms that integrate immunity in tissues with lymphatic networks, and potentially with immune organoids. Specifically, there have been significant advances in modeling the lymphatic vessels within the tumor immune microenvironment.^[^ ^55^, ^82^ ^]^ By seeding these lymphatic vessels with various tumor cell types, the platform enables systematic investigation of tumor‐induced lymphangiogenesis. Similarly, the effect of patient‐derived tumor‐derived fibroblasts on lymphatic sprouting, vessel permeability and lymphangiogenic genes was evaluated using a 3D microfluidic system.^[^ ^83^ ^]^ This was made using a collagen hydrogel with tumor‐associated fibroblasts co‐cultured with an LEC‐lined hollow channel. It was found that pro‐lymphangiogenesis phenotype correlated with higher tumor stage. On another note, a study of metastasis of cancer cells through the blood and lymphatic vasculature was performed by Cho et al.,^[^ ^84^ ^]^ where they developed an integrated system with using bioprinting and coculture with melanoma spheroids. Niec et al. developed a co‐culture of intestinal organoids in Matrigel over a monolayer of LECs revealing unique maintenance of intestinal stem cells by the LECs via Wnt signaling.^[^ ^85^ ^]^ Further advancements in the field involving disease and organ specific biophysical parameters and ECM components would make physiologically and pathologically distinct microenvironments more explorable.

Mazzagila et al.^[^ ^86^ ^]^ engineered a hydrogel‐based in vitro model that recapitulates key structural and functional features of murine lymph nodes (Figure 3D). By integrating primary murine immune cells with FRCs, LECs, and ECM components such as collagen and gelatin methacrylate (GelMA), this platform achieved spatial compartmentalization into distinct T and B cell zones alongside lymphatic conduit networks. Their findings highlight how stromal architecture and secreted factors shape immune cell dynamics within the lymph node microenvironment. Building on this, Morrison et al.^[^ ^87^ ^]^ developed an organotypic lymph node‐on‐chip model incorporating perfused, LEC‐lined microvessels to emulate human lymph node physiology (Figure 3C). This system fostered B cell enrichment and induced secretion of critical cytokines, including IL‐7 and CCL21, thereby supporting immune cell survival and directed migration. Together, these platforms demonstrate the potential of engineered lymphoid tissues to unravel the complex interplay between lymphatic structures and immune function.

## 7 Future Direction and Challenges

**Figure 4:**
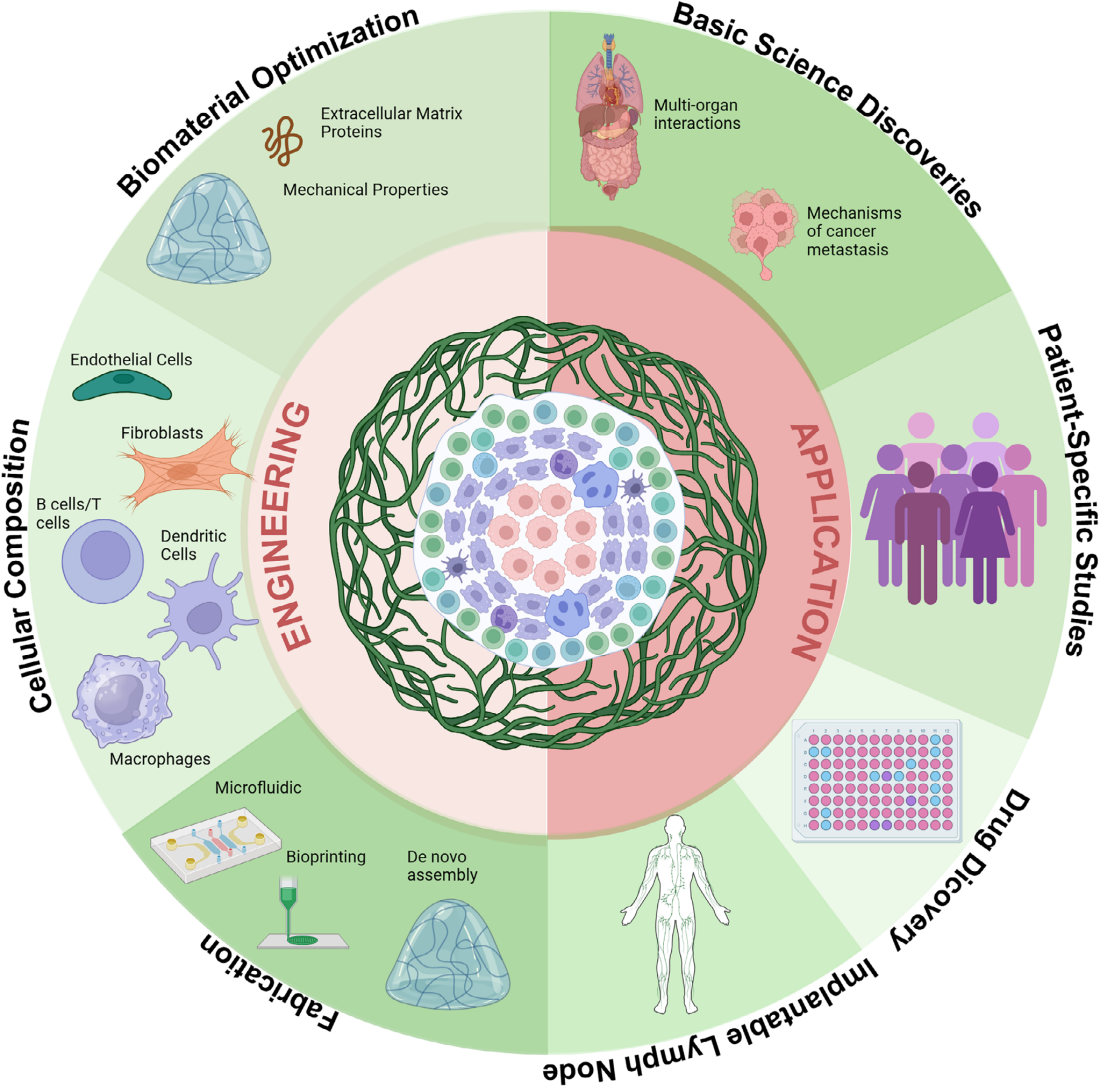
Schematic of an Integrated Immune Organoid Model. The engineering of an integrated immune organoid model can focus on three major areas: the biomaterials, cell types, and their sources, and fabrication techniques. Furthermore, the application of these integrated organoids will guide their engineering, too. These systems can be engineered for studying basic mechanisms of healthy and diseased tissues. They can be optimized to conduct patient‐specific responses using iPSC technologies, multi‐omics, and machine learning models. Similarly, high‐throughput systems incorporating tissue‐engineered constructs would enable better drug discovery. Lastly, an implantable construct can focus on long‐term survival and functionality in addition to biosafety. Made with Biorender.

Advancing the integration of lymphatic and immune organoids will require focused efforts across several key areas (**Figure** **4**). A critical step is the development of tunable, bioinspired extracellular matrix (ECM) platforms that better mimic native tissue environments. Current models often rely on limited ECM compositions, such as fibrin or collagen, which fail to capture the full biochemical and mechanical complexity of native tissues. Facile photopatterning techniques now enable the creation of perfusable microchannels within synthetic hydrogels, allowing precise spatial control over 3D vascular and lymphatic architectures. These advances recreate dynamic microphysiological environments with defined stiffness and biochemical gradients, essential for studying processes like fibrosis and tumor stiffening.^[^ ^88^, ^89^, ^90^, ^91^, ^92^ ^]^ Humanization of organoid platforms remains a major challenge. However, this is a non‐trivial task as it took our group 10 years to pioneer the synthetic biomaterials‐based human immune organoid,^[^ ^60^ ^]^ while our initial efforts focused on diligently developing and validating a murine immune organoid. Murine cells pose translational limitations due to interspecies differences. However, HLA matching between donor‐derived cells is critical to avoid allogeneic immune responses within these complex multicellular systems, posing an ongoing challenge for standardized platform development. Moving toward human primary cells or induced pluripotent stem cell (iPSC)‐derived stromal, endothelial, and immune populations will improve clinical fidelity and allow more accurate modeling of immune functions, including antigen‐specific memory and somatic hypermutation.^[^ ^93^ ^]^ Integration of lymphatic‐immune organoids into multi‐organ‐on‐chip systems represents a transformative opportunity to study inter‐organ crosstalk, immune surveillance, and systemic responses in real time. Linking lymph node models with upstream tissues such as skin, gut, or tumors will enable a comprehensive investigation of coordinated immune responses and disease mechanisms.

Standardization and scalability are essential for broader adoption. Developing open‐source protocols and automated culture systems will enhance reproducibility and enable high‐throughput applications in drug screening, immunotoxicology, and personalized medicine. Lastly, the incorporation of real‐time biosensors and live imaging tools will facilitate long‐term monitoring of immune behavior, cytokine dynamics, and metabolic activity. These capabilities are crucial for studying chronic immune responses, memory cell development, and the long‐term effects of immunotherapies or disease progression. Addressing these challenges will allow the next generation of lymphatic‐immune organoid systems to model human immunology and support therapeutic development more effectively.

Importantly, computational modeling across multiple scales holds significant potential to enhance the design and functionality of integrated immune organoids with lymphatic and vascular networks.^[^ ^94^ ^]^ Agent‐based in silico models can simulate cellular interactions based on fundamental mechanistic rules, enabling the prediction of immune and stromal cell behavior under varying biochemical and biophysical conditions. For example, the effects of modulating signaling molecule concentrations, such as VEGF‐C, can be systematically explored to inform optimal dosing strategies. Additionally, simulations of physiological forces within diverse geometric and microarchitectural contexts can guide the rational design of organoid structures to better emulate in vivo environments. Insights from developmental biology, such as the mechanisms underlying lymphatic valve formation, where localized cell accumulation acts as a nucleation site, can also be modeled computationally to test hypotheses and refine experimental designs prior to implementation. Finally, machine learning approaches integrating donor‐specific multi‐omics datasets hold promise for personalized organoid engineering. Predictive algorithms can recommend tailored hydrogel compositions, creating a “hydrogel atlas” that links biomaterial properties with immune phenotypes, enabling the fabrication of individualized microenvironments optimized for patient‐specific immune responses. In summary, advancing lymphatic‐immune organoid integration hinges on developing biomimetic ECM platforms, achieving humanization through compatible cell sources, and leveraging precise fabrication techniques like photopatterning to recreate dynamic microenvironments. Coupling these organoids with multi‐organ systems will enable comprehensive studies of immune function and inter‐organ communication. Complemented by computational modeling and machine learning, these efforts promise personalized, scalable platforms that faithfully recapitulate human immunity and accelerate therapeutic discovery.

## Conflict of Interest

A.J.G. and A.S. are co‐inventors on patent applications owned by the Georgia Tech Research Corp. on this general topic.
